# Machine Learning for Predicting Heart Failure Progression in Hypertrophic Cardiomyopathy

**DOI:** 10.3389/fcvm.2021.647857

**Published:** 2021-05-13

**Authors:** Ahmed S. Fahmy, Ethan J. Rowin, Warren J. Manning, Martin S. Maron, Reza Nezafat

**Affiliations:** ^1^Cardiovascular Division, Department of Medicine, Beth Israel Deaconess Medical Center and Harvard Medical School, Boston, MA, United States; ^2^Division of Cardiology, Hypertrophic Cardiomyopathy Center, Tufts Medical Center, Boston, MA, United States; ^3^Department of Radiology, Beth Israel Deaconess Medical Center and Harvard Medical School, Boston, MA, United States

**Keywords:** heart failure, hypertrophic cardiomyopathy, machine learning, risk factors, risk stratification

## Abstract

**Background:** Development of advanced heart failure (HF) symptoms is the most common adverse pathway in hypertrophic cardiomyopathy (HCM) patients. Currently, there is a limited ability to identify HCM patients at risk of HF.

**Objectives:** In this study, we present a machine learning (ML)-based model to identify individual HCM patients who are at high risk of developing advanced HF symptoms.

**Methods:** From a consecutive cohort of HCM patients evaluated at the Tufts HCM Institute from 2001 to 2018, we extracted a set of 64 potential risk factors measured at baseline. Only patients with New York Heart Association (NYHA) functional class I/II and LV ejection fraction (LVEF) by echocardiography >35% were included. The study cohort (*n* = 1,427 patients) was split into three disjoint subsets: development (50%), model selection (10%), and independent validation (40%). The least absolute shrinkage and selection operator was used to select the most influential clinical variables. An ensemble of ML classifiers, including logistic regression, was used to identify patients with high risk of developing a HF outcome. Study outcomes were defined as progression to NYHA class III/IV, drop in LVEF below 35%, septal reduction procedure, and/or heart transplantation.

**Results:** During a mean follow-up of 4.7 ± 3.7 years, advanced HF occurred in 283 (20% out of 1,427) patients. The model features included patients' sex, NYHA class (I or II), HCM type (i.e., obstructive or not), LV wall thickness, LVEF, presence of HF symptoms (e.g., dyspnea, presyncope), comorbidities (atrial fibrillation, hypertension, mitral regurgitation, and systolic anterior motion), and type of cardiac medications. The developed risk stratification model showed strong differentiation power to identify patients at advanced HF risk in the testing dataset (c-statistics = 0.81; 95% confidence interval [CI]: 0.76, 0.86). The model allowed correct identification of high-risk patients with accuracy 74% (CI: 0.70, 0.78), sensitivity 80% (CI: 0.77, 0.83), and specificity 72% (CI: 0.68, 0.76). The model performance was comparable among different sex and age groups.

**Conclusions:** A 5-year risk prediction of progressive HF in HCM patients can be accurately estimated using ML analysis of patients' clinical and imaging parameters. A set of 17 clinical and imaging variables were identified as the most important predictors of progressive HF in HCM.

## Summary

Heart failure (HF) progression is the most common adverse disease consequence in hypertrophic cardiomyopathy. However, identification of at-risk patients is currently limited and predominantly relies on identifying dynamic left ventricular outflow tract obstruction, which has limited specificity and does not allow for tailored treatment planning. A few recent studies investigated the prognostic value of individual HF risk factors (e.g., left ventricular function or longitudinal strain), each with limited sensitivity and specificity. To our knowledge, no study has reported a risk stratification model for progressive HF in HCM. In this study, we present a prediction model to identify individual HCM patients who are at high risk of developing advanced HF symptoms. Our model allows personalization of individual patients' clinical course and enables the potential development of future studies investigating earlier treatment in high-risk patients to determine if this can improve patient outcomes.

## Introduction

Hypertrophic cardiomyopathy (HCM) is the most common genetic heart disease with sudden cardiac death as the most visible and devastating consequence (1–4). Much attention has been placed on the identification of HCM patients at risk for sudden death, allowing for a mature sudden death risk stratification strategy that identifies the vast majority of at-risk individuals (3, 5). However, the most common adverse consequence of HCM is the development of advanced heart failure (HF) symptoms, occurring in 35–50% of patients and leading to substantial function disability and reduced quality of life (6–8).

The mechanism of exertional disability in HCM is predominantly secondary to dynamic left ventricular (LV) outflow tract (LVOT) obstruction occurring either at rest or with provocation, with these patients at higher risk for progressive symptoms (9–11), while nonobstructive patients are at substantially lower risk for symptom progression. However, risk stratification of patients based on the LVOT obstruction falls short of specificity needed for accurate disease management and treatment planning. For example, there is limited ability to stratify patients with LVOT obstruction who are at high risk for development of HF, as compared to those who survive to advance ages with no or mild symptoms. In contrast, nonobstructive HCM patients are considered at lower risk for development of advanced HF. However, medical therapy for patients with symptomatic nonobstructive HCM is limited and patients who develop advanced HF symptoms may ultimately require cardiac transplant as the only definitive treatment option (5, 9).

Few recent studies investigated the potential prognostic value of individual imaging and clinical parameters such as LV structural and functional parameters, cardiopulmonary exercise testing parameters, serum biomarkers, and global longitudinal strain (12–14). However, there is still a limited ability to predict HF progression in HCM and there is a need for a HF risk prediction model that allows more comprehensive evaluation of the patients' clinical parameters. Machine learning (ML) algorithms provide a powerful tool for learning complex relationships between the risk predictors and outcomes from a representative sample of the patients. ML-based models have been used to predict cardiovascular events with improved accuracy and generalizability compared to traditional risk predictors (15–19). Several studies showed that further improvement can be achieved by combining a number of ML models in an ensemble utilizing their versatile characteristics (15, 20, 21). In this study, we present an ML-based HF risk prediction model in HCM patients. To avoid arbitrarily selecting a specific ML model, we followed a systematic approach to build an ensemble of models that can learn the association between HF risk and clinical and imaging risk markers. We report the performance metrics of each individual model in the ensemble to illustrate the designing steps rather than providing a rigorous comparison of the different models.

## Materials and Methods

### Study Population and Outcome

The database of the HCM Institute at Tufts Medical Center (Boston, MA) containing data from 2,732 consecutive patients with HCM from June 2001 to Dec 2018 was interrogated. Data records for 880 patients (32%) with advanced HF symptoms at baseline (defined by New York Heart Association (NYHA) functional class III or IV) (*n* = 863), heart transplantation (*n* = 1), or septal reduction procedure (*n* = 11) or with LV ejection fraction by echocardiography <35% (*n* = 5) were excluded. Data on the most recent status of HF were obtained up to December 30, 2019, in 1,427 (77% of 1,852) patients by hospital visit or telephone contact with patients, family members, and referring physicians. Study outcomes were defined as progression in HF symptoms from NYHA functional classes I/II to classes III/IV, drop in LV ejection fraction to <35%, having underwent septal reduction procedure, or having had (or added to the waiting list of) heart transplantation during follow-up. The mean ± SD follow-up duration from initial clinical evaluation at the Tufts Medical Center to the earliest of progression to class III/IV date or most recent contact was 4.7 ± 3.7 years. The average time to advanced HF symptoms in our cohort was 2.7 ± 2.6 years. The clinical diagnosis of HCM was based on two-dimensional transthoracic echocardiographic identification of otherwise unexplained hypertrophied non-dilated LV (wall thickness ≥13 mm) (3, 22). Patients had been referred for targeted subspecialty evaluations, including diagnosis, risk stratification, and treatment. Patients with phenocopies of HCM (e.g., Fabry disease, LAMP2 cardiomyopathy, PRKAG2, or amyloidosis) were excluded. This study was approved by the institutional review board at Tufts Medical Center, allowing a retrospective review of medical records and granting a waiver of informed consent in accordance with 45 CFR 46.116(d).

### Potential Risk Predictors

The model was built using potential clinical, demographic, and imaging risk markers (*n* = 64; Supplementary Table 1) measured at the time of initial patient evaluation including (1) baseline demographics (e.g., age and sex); (2) HF risk factors (e.g., symptoms of fatigue, dyspnea, and syncope); (3) imaging data (e.g., echocardiography LV ejection fraction, LA size, and maximum wall thickness); (4) cardiac medications (e.g., beta blocker and calcium channel blocker); and (5) comorbidities (e.g., hypertension, atrial fibrillation, stroke, and implantable cardiac device). A risk factor representing obstructive (or non-obstructive) HCM was defined by a LV outflow tract (LVOT) gradient ≥30 mmHg at rest or with provocation (i.e., exercise or Valsalva maneuver). Nonobstructive HCM was identified by a LVOT gradient <30 mmHg both at rest and with provocation. Categorical variables were replaced by an integer ranging from 0 to the maximum number of categories (as indicated in Supplementary Table 1). Variables with >5% missing data were not included. Missing measurements of the included variables were imputed using the *k*-nearest neighbor method, with *k* set to 1 to preserve the original variability in data distribution (23).

**Figure 1:**
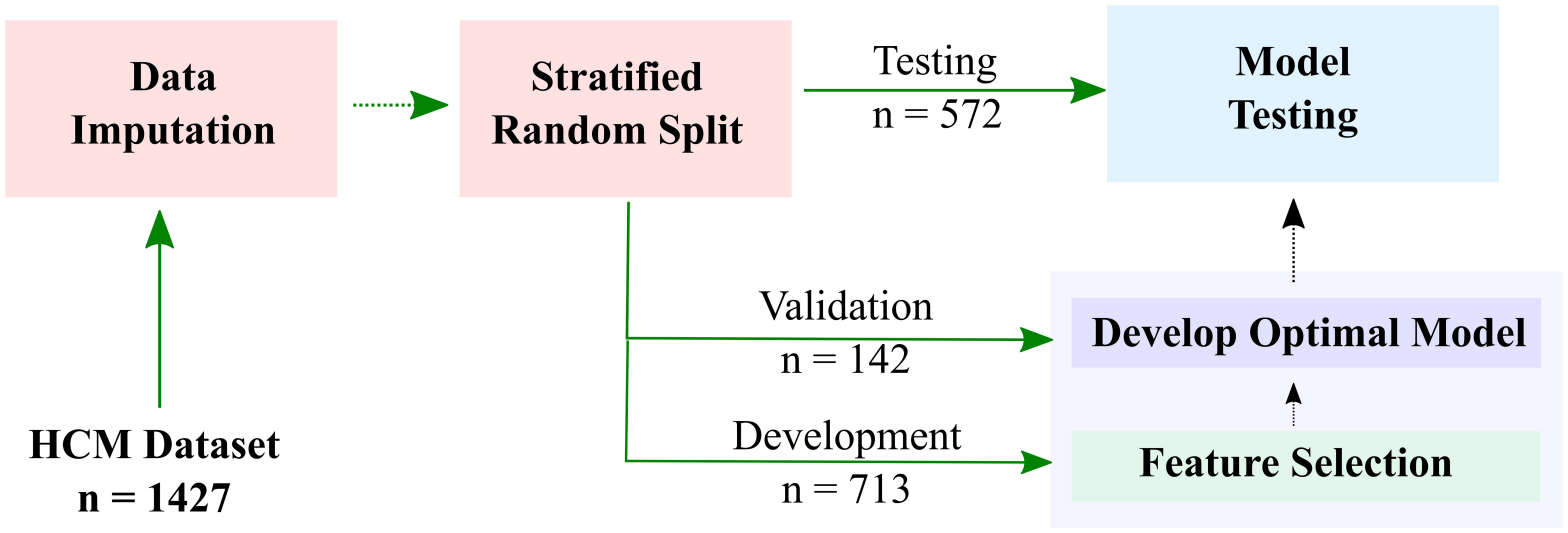
Workflow of developing a machine learning-based model for predicting risk of heart failure (HF) in hypertrophic cardiomyopathy (HCM) patients. Datasets are imputed and split into development (50%), model-selection (10%), and independent-validation (40%) subsets. The development subset is used to select the most important features, and the validation subset is used for model optimization. The final HF stratification model is then used to predict HF risk in the testing subset.

For the purpose of developing the HF risk model, the patients were split into three subsets (Figure 1): (1) development subset (713 patients (50%); (2) model-selection subset (142 patients (10%); and (3) independent-validation subset (572 patients (40%). Stratified random sampling was used to split the data such that the ratio of positive to negative HF outcomes was the same in all subsets.

### Risk Predictor Selection

The set of most important clinical variables was selected using the least absolute shrinkage and selection operator (LASSO) (24). To determine the optimal number of features, LASSO feature selection was repeated to select the best *k* features (with *k* ranging from 1 to 40). For each value of *k*, a logistic regression model was developed and evaluated using a 10-fold cross-validation scheme. In this scheme, the development dataset is split into 10 disjoint subsets, where nine subsets were used for training the model and one subset is used for model evaluation. The process was repeated 10 times to try all possible 10 different selections of training-evaluation subsets. The average model performance [measured by the area under the curve (AUC) of the receiving operating characteristics (ROC), or c-statistics] over the 10 repetitions was used to determine the optimal number, *k*, and specify the most important clinical variables.

### Model Selection

The development subset was used to train and optimize six different state-of-the-art ML classifiers: logistic regression (LG), random forests (RF), support-vector machines (SVM), gradient boosted decision trees (GBC), adaptive boosted decision trees (ADB), and neural networks (NN). Ten-fold cross-validation was used to determine the optimal model parameters. Each resulting model was then evaluated using the model-selection subset (142 patients) to determine the best model. An ensemble of the three best-performing models was used as the final HF risk stratification model. The outputs of models comprising the ensemble were merged using logistic regression. The final ensemble output was a normalized probability value (i.e., from 0 to 1) representing the patient's risk to develop HF outcome.

### Model Testing and Performance Evaluation

The final optimal models were used to predict the HF risk for the patients in the independent validation dataset. The models output a value representing the probability that a patient develops advanced HF symptoms within a 5-year follow up interval. We used AUC (or c-statistics) to estimate the discriminatory power of the model to identify patients at risk of progressive HF. An arbitrary operating point represented by a probability of 50% was used to identify patients at high risk of HF and used to compute the F1 score, sensitivity, specificity, and accuracy of each model. The contribution of each input variable to the model output for each patient (i.e., probability of developing progressive HF) was assessed by the Shapley values (25). Shapley values approximate the impact of removing the variable on the model prediction while taking into account the interactions among all variables. Model development was done using Python-V3.7 (Python Software Foundation, Fredericksburg, VA) and Scikit-learn Ver-0.23.2 (scikit-learn.org) on a PC with Quadro K620 graphics processing unit (Nvidia, Santa Clara, CA). For Shapley value computations, we used the SHapley Additive exPlanations (SHAP) analysis library (26). The final model is available at https://doi.org/10.7910/dvn/ffnlpe for external validation by other researchers.

### Statistical Data Analyses

Data are displayed as mean ± SD for continuous variables and as proportions for categorical variables. The Student (two-sample) *t*-test was used to assess statistical significance for continuous variables and *z*-test for comparing population proportions. AUC, sensitivity, specificity, and average F1 score were used to evaluate the model performance. Parametric estimation for the variance was used to compute the 95% confidence interval (CI), and a *p* ≤ 0.05 was considered significant (reported as two-sided). Statistical calculations were performed with the Matlab statistical toolbox (version R2018b, Mathworks, Natick, MA).

## Results

**Figure 2:**
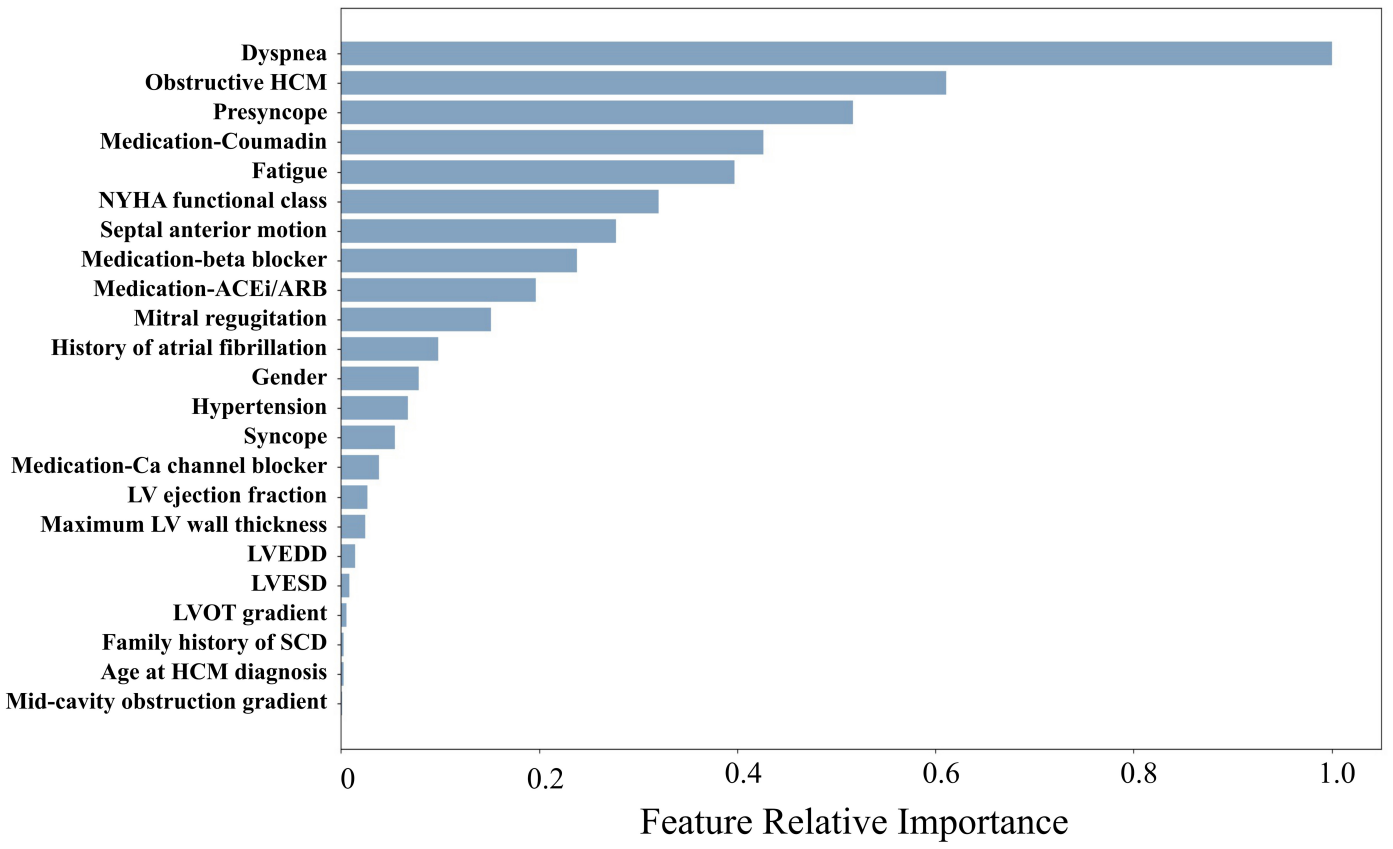
Relative importance scores for the risk factors included in the study (only factors with nonzero scores are displayed).

**Figure 3:**
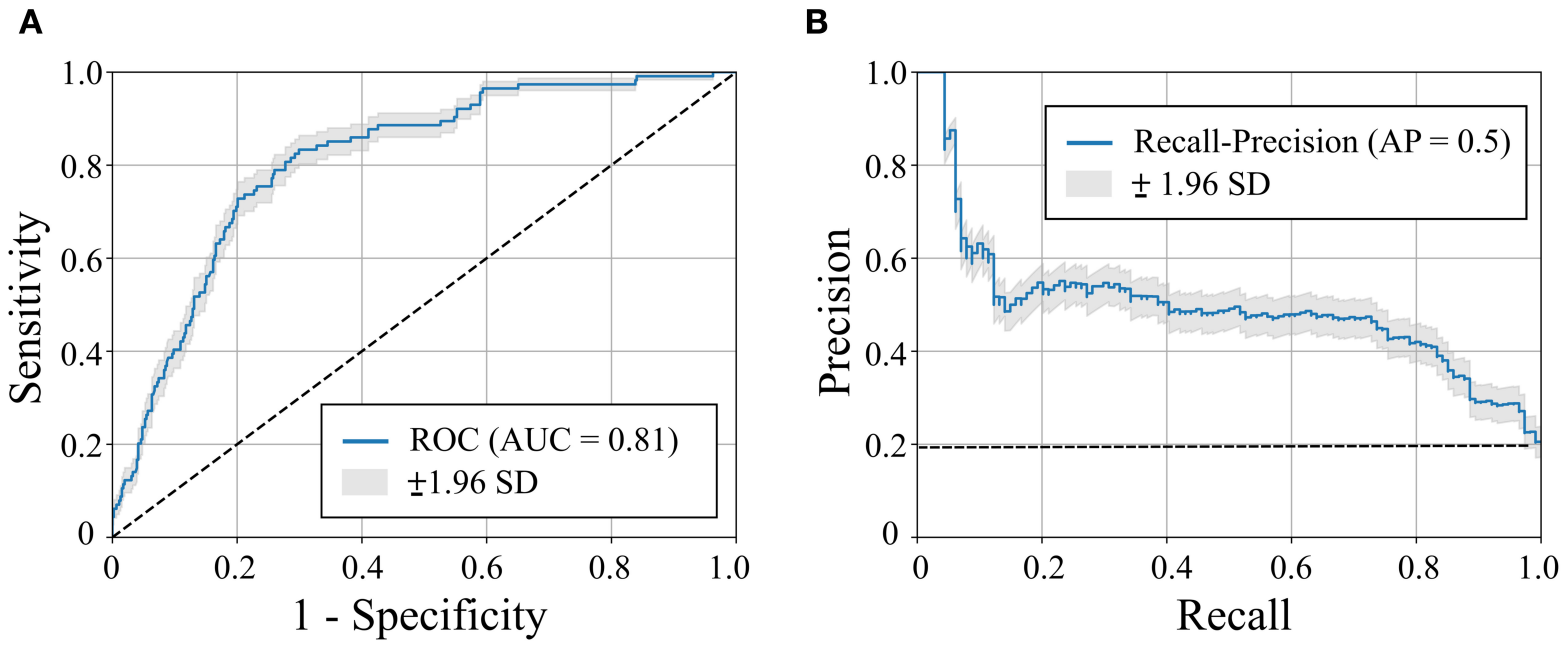
Receiver operating characteristic (ROC) curve **(A)** and recall-precision curve **(B)** for the machine learning-based heart failure (HF) risk stratification in hypertrophic cardiomyopathy patients (*n* = 572). Dashed line represents pure-chance stratification AUC = 0.5 in **(A)** or precision = ratio of HF outcomes in the dataset (=20%) **(B)**. AUC = area under the curve. AP, average precision; SD, standard deviation.

The mean age of the patients included in this study (*n* = 1,427; 69% men) was 52 ± 17 years with a mean follow-up time of 4.7 ± 3.6 years (median 3.7 years). The baseline characteristics of the patient cohort are shown in Table 1. Twenty-three features (of 64) showed a non-zero importance score using LASSO feature selection analysis (Figure 2). The optimal number of important features that maximized HF risk stratification performance (c-statistics) in the development subset was 17 features (Table 1). Four classifiers yielded the highest three AUC scores: LG (0.79), GBC (0.79), NN (0.78), and SVM (0.78) (Table 2). An ensemble of LG-GBC-SVM was used as the final prediction model. The final model showed strong power to differentiate low- from high-risk patients in the testing subset (572 patients) with AUC = 0.81 [95% CI: 0.76–0.86] (Figure 3). The model showed accuracy of 74% [95% CI: 0.70–0.78], sensitivity of 80% [95% CI: 0.77–0.83], and specificity of 72% [95% CI: 0.68–0.76] (Table 3). The model performance metrics for the different age and sex subgroups was comparable and showed overlapped 95% CI, as indicated in Table 3. SHAP analysis showed that obstructive HCM and NYHA functional class II were associated with higher risk compared to non-obstructive HCM and NYHA functional class I (Figure 4). Also, presence of HF symptoms (dyspnea, fatigue, syncope, and presyncope) or abnormal heart function or structure (e.g., reduced LV ejection fraction, increased wall thickness, septal anterior motion, and mitral regurgitation) increased the risk of developing progressive HF. Also, three cardiac medications (Coumadin, beta blockers, and calcium channel blockers) showed an association with increased HF risk while the angiotensin-converting enzyme inhibitor (ACEi) or angiotensin-receptor blocker (ARB) was associated with low HF risk. Additionally, risk of progressive HF was higher in males and patients with history of atrial fibrillation and/or without hypertension (Figure 4).

**Table 1:** Baseline clinical characteristics for the hypertrophic cardiomyopathy (HCM) patients at initial clinical assessment.

	**Model input**	**ALL (*n* = 1,427)**	**HF– (*n* = 1,144)**	**HF+ (*n* = 283)**	***p*-value**
Male, *n* (%)	Yes	985 (69)	818 (72)	167 (59)	<0.001
Age at HCM diagnosis (years), mean ± SD (median)	No	45 ± 18 (48)	45 ± 18 (48)	46 ± 18 (48)	0.55
NYHA functional class	Yes				
I, *n* (%)		794 (56)	733 (64)	61 (22)	<0.001
II, *n* (%)		633 (44)	411 (36)	222 (78)	<0.001
Family history of HCM, *n* (%)	No	369 (26)	296 (26)	73 (26)	0.98
Family history of sudden death secondary to HCM, *n* (%)	No	154 (11)	41 (4)	28 (10)	0.58
Family history of end-stage HCM, *n* (%)	No	41 (3)	31 (3)	10 (4)	0.49
Obstructive HCM, *n* (%)	Yes	747 (52)	525 (45)	229 (81)	<0.001
LV outflow tract gradient (mmHg), mean ± SD (median)	No	19 ± 5 (17)	15 ± 32 (0)	34 ± 41 (0)	<0.001
Mid-cavity LV obstruction gradient (mmHg), mean ± SD (median)	No	3 ± 12 (0)	3 ± 12 (0)	2 ± 12 (0)	0.52
Maximum LV wall thickness (mm), mean ± SD (median)	Yes	19 ± 5 (17)	18 ± 4 (17)	20 ± 5 (19)	<0.001
LV ejection fraction (%), mean ± SD (median)	Yes	64 ± 5 (65)	63 ± 5 (65)	64 ± 6 (65)	0.29
LV EDD (mm), mean ± SD (median)	No	42 ± 7 (42)	42 ± 7 (42)	41 ± 7 (41)	<0.001
LV ESD (mm), mean ± SD (median)	No	27 ± 6 (26)	27 ± 6 (26)	26 ± 5 (25)	0.002
LV apical aneurysm, n (%)	No	42 (3)	40 (4)	2 (1)	<0.001
LA diameter (mm), mean ± SD (median)	No	40 ± 7 (40)	40 ± 7 (40)	42 ± 7 (41)	0.001
Systolic anterior motion, *n* (%)	Yes	927 (68)	681 (63)	246 (89)	<0.001
Mitral regurgitation, *n* (%)	Yes	562 (39)	410 (36)	152 (54)	<0.001
NSVT seen on ambulatory monitor, *n* (%)	No	137 (10)	120 (26)	17 (6)	0.008
Syncope, *n* (%)	Yes	139 (10)	100 (9)	37 (13)	0.046
Fatigue, *n* (%)	Yes	198 (14)	125 (11)	73 (26)	<0.001
Presyncope, *n* (%)	Yes	71 (5)	47 (4)	24 (8)	0.014
Dyspnea, *n* (%)	Yes	645 (45)	417 (39)	226 (80)	<0.001
Hypertension, *n* (%)	Yes	461 (32)	379 (33)	82 (29)	0.17
Atrial fibrillation, *n* (%)	Yes	203 (14)	158 (14)	51 (18)	0.24
Patients with ICD placed prior to initial visit, *n* (%)	No	159 (11)	117 (10)	42 (15)	0.045
Appropriate ICD therapy prior to initial visit, *n* (%)	No	17 (1)	11 (1)	6 (2)	0.20
Resuscitated cardiac arrest prior to initial visit, *n* (%)	No	24 (2)	19 (2)	5 (2)	0.91
Medications—beta blocker, *n* (%)	Yes	807 (57)	610 (53)	197 (70)	<0.001
Medications—calcium channel blocker, *n* (%)	Yes	290 (20)	212 (19)	78 (28)	0.002
Medications—ACEi/ARB, *n* (%)	Yes	309 (22)	266 (23)	43 (15)	0.001
Medications—coumadin, *n* (%)	Yes	80 (6)	56 (5)	24 (8)	0.044

*Data represents n (%) or mean ± SD (median). HF+, patients developed heart failure during follow-up (i.e., positive HF outcome); HF–, patients without HF outcome; ACEi, angiotensin-converting enzyme inhibitor; ARB, angiotensin-receptor blockers; ICD, implantable intracardiac defibrillator; LA, left atrium; LV, left ventricle; EDD, end diastolic diameter; ESD, end systolic diameter; LVOT, left ventricular out flow tract; NSVT, non-sustained ventricular tachycardia; NYHA, New York Heart Association; SD, standard deviation*.

**Table 2:** Performance evaluation of the different machine learning models using the model-selection dataset (143 patients; 28 positive heart failure outcomes).

**Classifier type**	**AUC**	**ACC**	**Sn**	**Sp**	**F1 score**
Neural networks (NN)	0.78	0.68	0.82	0.65	0.64
Support vector machines (SVM)	0.78	0.69	0.75	0.68	0.63
Random forests	0.67	0.70	0.21	0.93	0.59
Gradient boosted DT (GBC)	0.79	0.69	0.64	0.70	0.62
Adaptive boosted DT	0.77	0.79	0.14	0.95	0.54
Logistic regression (LG)	0.79	0.71	0.71	0.71	0.65
LG + GBC + NN	0.79	0.71	0.71	0.71	0.65
LG + GBC + SVM	0.80	0.71	0.71	0.71	0.65

*DT, decision trees; AUC, area under the receiver operating characteristic curve; ACC, accuracy; Sn, sensitivity; Sp, specificity*.

**Table 3:** Performance evaluation of the ensemble model using the independent-validation dataset.

	**AUC**	**ACC**	**Sn**	**Sp**	**F1 score**
**All patients** (n^*^= 572; 114 HF+)	0.81 [CI: 0.76–0.86]	0.74 [CI: 0.70–0.78]	0.80 [CI: 0.77–0.83]	0.72 [CI: 0.68–0.76]	0.68 [CI: 0.64–0.72]
**Female** (*n* = 188; 55 HF+)	0.76 [CI: 0.68–0.84]	0.69 [CI: 0.62–0.76]	0.80 [CI: 0.74–0.86]	0.64 [CI: 0.57–0.71]	0.67 [CI: 0.60–0.74]
**Male** (*n* = 384; 59 HF+)	0.81 [CI: 0.74–0.88]	0.76 [CI: 0.72–0.80]	0.75 [CI: 0.71–0.79]	0.76 [CI: 0.72–0.80]	0.66 [CI: 0.61–0.71]
**Age^#^:** **<** **20 years** (*n* = 76; 14 HF+)	0.78 [CI: 0.63–0.93]	0.82 [CI: 0.73–0.91]	0.71 [CI: 0.61–0.81]	0.84 [CI: 0.76–0.92]	0.73 [CI: 0.63–0.83]
**Age: 20–40 years** (*n* = 139; 26 HF+)	0.84 [CI: 0.74–0. 94]	0.74 [CI: 0.67–0.81]	0.85 [CI: 0.79–0.91]	0.72 [CI: 0.65–0.79]	0.68 [CI: 0.60–0.76]
**Age: 40–60 years** (*n* = 229; 46 HF+)	0.81 [CI: 0.73–0.89]	0.72 [CI: 0.66–0.78]	0.85 [CI: 0.80–0.90]	0.69 [CI: 0.63–0.75]	0.68 [CI: 0.62–0.74]
**Age:** **≥** **60 years** (*n* = 128; 27 HF+)	0.77 [CI: 0.66–0.88]	0.65 [CI: 0.57–0.73]	0.78 [CI: 0.71–0.85]	0.61 [CI: 0.53–0.69]	0.61 [CI: 0.53–0.69]

* *n represents number of patients (of 572 patients in the testing subset)*.

# *Age at diagnosis of hypertrophic cardiomyopathy. HF+, positive heart failure outcomes; CI: 95% confidence interval; AUC, area under receiver operating characteristic curve; ACC, accuracy; Sn, sensitivity; Sp, specificity*.

**Figure 4:**
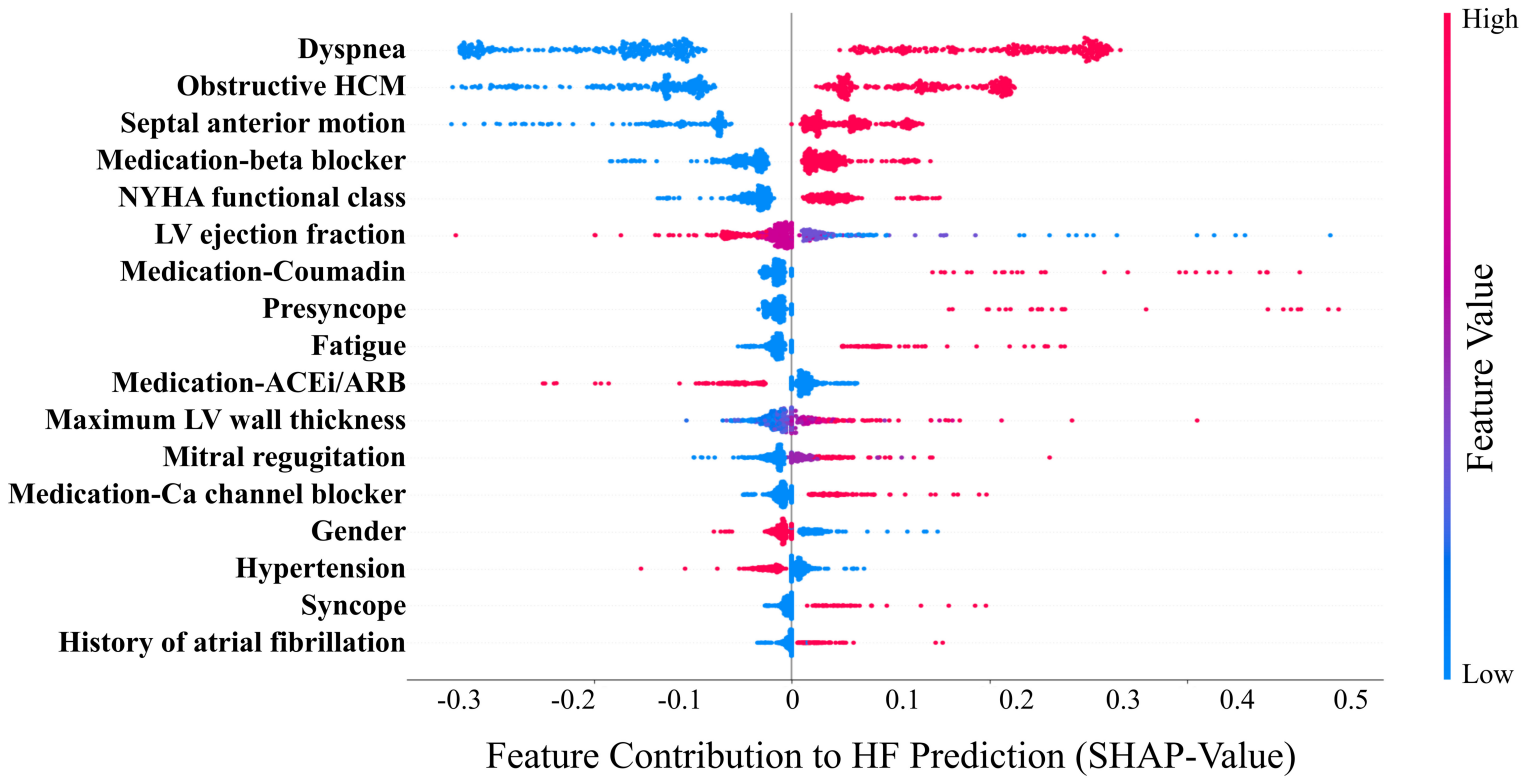
Relative contribution (SHAP-values) of the model variables (*n* = 17) to heart failure (HF) prediction. Each point in the graph indicates the contribution of the corresponding clinical variable to the HF prediction of one patient. Ca, calcium; ACEi, angiotensin-converting enzyme inhibitor; ARB, angiotensin-receptor blockers. HCM, hypertrophic cardiomyopathy; LV, left ventricle; NYHA, New York Heart Association.

## Discussion

We present an ML-based study to develop and test a prediction model for progressive HF in HCM. There has previously been limited ability to predict HF risk in HCM as a number of disease features appear to impact symptom progression limiting accuracy of traditional prediction models. In our study, an ensemble of machine learning classifiers, including logistic regression, is used to accurately predict the risk of progressive HF over an average of a 5-year follow-up period. The most significant variables in our models included clinical and imaging variables that have previously been individually linked to progressive HF in HCM, but with limited accuracy. Thereby, the ability to predict progressive HF symptoms appears to be related to an interaction of these variables. We initially included all 64 measured risk factors to determine if specific symptoms (e.g., dyspnea, fatigue, or chest pain) were predictive of the development of advanced HF over time. This allowed the final model to include risk factors that are not completely independent. For example, both dyspnea and NYHA class were significant factors in the model. While dyspnea is included as part of NYHA class evaluation, notably a number of other factors ultimately play into the determination of NYHA class (e.g., degree of effort leading to dyspnea and degree of fatigue with exertion). In our cohort, 68 patients with dyspnea were in NYHA class I while 56 patients without dyspnea were in NYHA class II. All machine learning techniques studied in this work, except random forests, showed comparable accuracy (77–79%) for predicting the endpoints. An ensemble of the three best models showed a slightly higher accuracy (80%). Although the study endpoints included LV ejection fraction depression and cardiac transplantation, the small number of events during our follow-up period (*n* = 3 and 1, respectively) does not allow separate prediction of these events. Prediction of these events separate from progression of the NYHA class requires longer follow-up periods and a larger patient cohort to account for the low incidence rate of these events.

Our results demonstrate that the model performance is comparable in male and female patients. Also, there was no statistical significance in performance among the different age groups. However, the model average discriminating power, measured by AUC, was relatively high (≥0.81) in patients within the 20–60-year-old groups compared to the other two groups. This may be explained by the generally high representation of patients in this age range in our dataset (62%). We also note that the limited number of positive events in the youngest age group does not allow reliable prediction of HF, which was indicated by the wide 95% CI.

Progressive and advanced HF development is the most common adverse pathway in HCM. With the availability of mature strategy for identification of patients at risk for sudden death and utilization of ICDs for sudden death prevention, HF has become the most common cause of HCM death. While most HCM patients will have a benign clinical course without HF progression, there has been an inability to identify at-risk patients, leading to uncertainty from treating clinicians as to which patients are in need for more aggressive therapy and closer clinical follow-up. Similarly, there has been uncertainty for patients regarding their disease-related natural history and individual risk. The present model allows for clarification of an individual risk and allows for a more personalized treatment approach regarding both need for closer clinical follow-up and more aggressive treatment. For example, the model can identify individual patients who may develop advanced HF with relatively high sensitivity (80%) and specificity (72%). This can open the opportunity for adopting more aggressive treatment to improve clinical outcomes in higher-risk individuals and closer follow-up. Meanwhile, it can offer a substantial reassurance that low-risk patients are unlikely to need interventional procedures over a 5-year period. However, we note that the presented model is developed based on a 5-year follow-up period and may not be accurate to predict HF beyond 5 years. The lack of established HF stratification models in HCM does not allow benchmarking of our model. However, we note that the stratification power and accuracy of our model are comparable to those reported for established sudden cardiac death risk stratification models (27–29).

While the impact of medical therapy to change the natural history of HCM remains controversial without data to routinely support implementation (9, 30), a more targeted approach to initiation of medical therapy specifically in patients identified at higher risk is deserving of a further study. This is particularly relevant given the ongoing research into novel therapeutic interventions in HCM, including myosin modulators which may prove more powerful treatments to alter HCM phenotype and prevent disease progression (31).

Our study has a number of limitations. First, our HF prediction model is designed to accommodate a typical clinical protocol implemented by a single medical center and is not tested using data acquired using different protocols. Also, given the longitudinal nature of our cohort with patients seen and evaluated over a 15-year period, more novel potential risk markers, such as serum biomarkers or mechanical deformation parameters such as global longitudinal strain (12), are not available but may offer additional dimensions to the model. Additionally, not every patient in this study was followed for the full 5-year term and patients who did not develop HF symptoms during the follow-up period were treated as not having the outcome of interest, which could bias the model.

In conclusion, our machine learning model allowed for accurate identification of HCM patients at risk for HF progression within a 5-year follow-up period. The model is based on 17 significant risk factors including imaging parameters (e.g., LVOT obstruction, septal anterior motion, and LV ejection fraction), cardiac medications (e.g., beta-blockers and coumadin), and physical symptoms of heart failure (e.g., dyspnea and fatigue). This may allow personalization of individual patients' clinical course into clinical practice and closer clinical follow-up in high-risk individuals. In addition, the developed models allow the opportunity for future research on implementation of earlier disease-specific treatment in high-risk patients to determine if this can prevent symptom progression and improve outcomes.

## Data Availability Statement

The data analyzed in this study was subjected to the following licenses/restrictions: Participant data used in this study are not publicly available at present. The source code of the machine learning algorithm implementation and the final (trained) model was available at https://doi.org/10.7910/dvn/ffnlpe.

## Ethics Statement

The studies involving human participants were reviewed and approved by the institutional review board at Tufts Medical Center, allowing retrospective review of medical records and granting a waiver of informed consent in accordance with 45 CFR 46.116(d). Written informed consent from the participants' legal guardian/next of kin was not required to participate in this study in accordance with the national legislation and the institutional requirements.

## Author Contributions

MM and RN: guarantor of integrity of entire study. ER and MM: data acquisition. AF: algorithm implementation. AF and ER: statistical analysis. AF, ER, and RN: literature research. ER, WM, MM, and RN: clinical studies. Underlying data was accessed and verified by AF, ER, and RN. All authors conceptualization and formulation of study design and overall goals, data curation and analysis/interpretation, manuscript drafting, editing, or manuscript revision for important intellectual content, approval of final version of submitted manuscript, and agreement to ensure any questions related to the work are appropriately resolved.

## Conflict of Interest

The authors declare that the research was conducted in the absence of any commercial or financial relationships that could be construed as a potential conflict of interest.

## Abbreviations

ADB: Adaptive boosted decision trees classifier
AUC: Area under curve of the receiving operator characteristics
CI: 95% Confidence interval
CMR: Cardiovascular magnetic resonance imaging
GBC: Gradient boosted decision trees classifier
HCM: Hypertrophic cardiomyopathy
HF: Heart failure
LA: Left atrium
LG: Logistic regression classifier
LV: Left ventricle
LVOT: Left ventricle outflow tract
ML: Machine learning
NYHA: New York Heart Association
NN: Neural networks
RF: Random forests
SVM: Support-vector machine.
