# Supplementary material for: Machine Learning for Predicting Heart Failure Progression in Hypertrophic Cardiomyopathy
Source: Front Cardiovasc Med. 2021 May 13;8:647857. doi: 10.3389/fcvm.2021.647857 (PMC8155292; doi:10.3389/fcvm.2021.647857)
Supplement: Supplementary file 1 [file Table_1.DOCX]

**Supplementary Table S1:** All clinical variables (n = 64) extracted from electronic health records for 1427 patients and used for model development.

| **Clinical Variable** | **Measurements**^#^ |
| --- | --- |
| **Gender** | 985(69) |
| **Age at HCM diagnosis** | 45±18(48) |
| **Obstructive HCM** | 747(52) |
| **Massive hypertrophy** | 84(6) |
| **Non-sustained ventricular tachycardia seen on holter** | 137(10) |
| **Syncope** | 137(10) |
| **Dyspnea** | 645(45) |
| **Chest pain** | 252(18) |
| **Fatigue** | 198(14) |
| **Presyncope** | 71(5) |
| **Palpitations** | 192(14) |
| **NYHA functional class** | 1-2(1) |
| **Implantable cardioverter device (ICD)** | 159(11) |
| **Appropriate ICD shocks for VT/VF prior to initial visit** | 17(1) |
| **Number of ICD shocks** | 0-8(0) |
| **Permanent pace maker** | 21(1) |
| **Mitral valve surgery** | 2(0) |
| **VT ablation** | 4(0) |
| **Coronary artery bypass graft** | 6(0) |
| **Stents** | 36(3) |
| **Cardioversion** | 64(4) |
| **Number of DC cardioversions** | 0-4(0) |
| **Atrial fibrillation ablation** | 16(1) |
| **Number of Atrial fibrillation ablations** | 0-3(0) |
| **Recurrent atrial fibrillation after Ablation** | 13(1) |
| **Atrial fibrillation** | 199(14) |
| **Resuscitated cardiac arrest prior to initial visit** | 24(2) |
| **Hypertension** | 461(32) |
| **Coronary artery disease** | 79(6) |
| **Prior myocardium infarction** | 22(2) |
| **Stroke** | 31(2) |
| **Type of stroke** | 0-2(0) |
| **Family history of SCD** | 154(11) |
| **Family history of SCD: relation to patient** | 0-4(0) |
| **Family history of SCD: multiple relatives** | 54(4) |
| **Family history of HCM** | 369(26) |
| **Family history of end stage HCM** | 41(3) |
| **Family history of heart transplant due to HCM** | 26(2) |
| **Medications-Beta blocker** | 807(57) |
| **Medications-Calcium channel blockers** | 290(20) |
| **Medications-Disopyramide** | 20(1) |
| **Medications-ACE inhibitor or ARB** | 309(22) |
| **Medications-Spironolactone** | 16(1) |
| **Medications-Diuretic (including HCTZ/loop diuretics)** | 151(11) |
| **Medications-Amiodarone** | 27(2) |
| **Medications-Coumadin** | 80(6) |
| **Medications-Aspirin** | 405(28) |
| **Medications-Statin** | 459(32) |
| **Medications-Novel anti-coagulation*** | 51(4) |
| **Medications-Other anti-arrhythmic**** | 44(3) |
| **Medications-Other cardiac medications***** | 38(3) |
| **Maximum LV wall thick (mm)** | 19±5(17) |
| **Septal anterior motion** | 927(68) |
| **LVOT gradient (mmHg)** | 19±35(0) |
| **Mid-Cavity obstruction gradient** | 3±12(0) |
| **Mitral regurgitation** | 0-4(0) |
| **LV ejection fraction (%)** | 64±5(65) |
| **LA diameter (mm)** | 40±7(40) |
| **LV end diastolic diameter (mm)** | 42±7(42) |
| **LV end systolic diameter (mm)** | 27±6(26) |
| **Severe aortic stenosis** | 9(1) |
| **Apical HCM** | 161(11) |
| **Apical aneurysm** | 42(3) |
| **End-stage HCM** | 25(2) |

^#^Values are mean±SD (median), n (%), or min-max (median) for all patients.

^*^ Sotalol, Dronedarone, Flecainide, Procainamide, Digoxin, Mexiletine, Dofetilide.

^**^ Eliquis, Xarelto, Pradaxa, Lovenox,

^***^ Milrinon, Nitroglycerin, Clonidine, Nitrates, Ranexa, Imdur, Ranolazine, Eplerenone.
